# Heterologous expression and characterization of a new lipase from *Pseudomonas fluorescens* Pf0–1 and used for biodiesel production

**DOI:** 10.1038/s41598-017-16036-7

**Published:** 2017-11-16

**Authors:** Wu Liu, Menggang Li, Yunjun Yan

**Affiliations:** Key Laboratory of Molecular Biophysics, Ministry of Education, College of Life Science and Technology, Huazhong University of Science and Technology, Wuhan, 430074 China

## Abstract

As a kind of important biocatalysts, *Pseudomonas* lipases are commonly applied in various industrial fields. *Pflip1*, a new extracellular lipase gene from *Pseudomonas*. *fluorescens* Pf0–1, was first cloned and respectively expressed in *Escherichia coli* BL21(DE3) and *Pichia pastoris* KM71, the recombinant proteins Pflip1a and Pflip1b were later purified separately. Then Pflip1a was further characterized. The optimum pH of Pflip1a was 8.0 and its optimal temperature was 70 °C. After incubation at 70 °C for 12 h, Pflip1a could retain over 95% of its original activity. It showed the highest activity towards *p*-nitrophenyl caprylate. Moreover, its activity was profoundly affected by metal ion, ionic surfactants and organic solvents. Furthermore, the two obtained recombinant lipases were immobilized on the magnetic nanoparticles for biodiesel preparation. The GC analysis showed that for the immobilized lipases Pflip1b and Pflip1a, the biodiesel yield within 24 h respectively attained 68.5% and 80.5% at 70 °C. The activities of the two immobilized lipases still remained 70% and 82% after 10 cycles of operations in non-solvent system. These characteristics and transesterification capacity of the recombinant protein indicated its great potential for organic synthesis, especially for biodiesel production.

## Introduction

Lipases, also known as triacylglycerol acylhydrolase (EC 3.1.1.3), are a big kind of hydrolases which act on carboxylic ester bonds, and completely hydrolyze triglycerides into fatty acids and glycerol. Additionally, they can catalyze esterification and transesterification reactions in non-aqueous media. These features make lipases play a significant role in biotechnological industries^1–4^. Lipases can be easily isolated from animals, plants and microorganisms, while bacteria, yeast and fungi are the main sources of the commercial lipases^5^. Bacterial lipases exhibit higher thermostability and tolerance to denaturing reagents and/or organic solvents in comparison with fungal and yeast lipases, and they are also more amenable to genetic manipulation^6^. So, they are widely applied in industry and academic researches. Among various bacterial lipases, those from *Pseudomonas* and *Burkholderia* (formerly *Pseudomonas*) genera are the commonest members applied in the field of biotechnological applications^7^. Particularly, it has been proved that lipase(s) from *Pseudomonas fluorescens* possess the enzymatic merits in chiral resolution of racemic mixtures and biodiesel production^8–10^.

In general, lipases from *Pseudomonas* have be classified into two categories. Lipases derived from *P*. *aeruginosa* and *B*. *glumae* belonging to one category. The functional expression of such enzymes requires a lipase-specific foldase (Lif). These enzymes provide classic signal peptide to complete their secretion through cell membrane. And lipases from *P*. *fluorescens* and *Serratia marcescens* were as the members of the other category. This sorts of lipases do not contain typical N-terminal signal peptide while containing C-terminal-targeting signal. Their secretion is completed via ATP-binding cassette (ABC) exporters which consist of an inner membrane ATP-binding cassette, a membrane fusion protein and an outer membrane protein^11,12^.

Because of their function in the degradation of fats and synthesis of esters, lipases have extensive applications, for instance, processing of fats, detergents, food processing, synthesis of fine chemicals, paper manufacturing, production of maquillage, and medicines^13^. As known, efficiently expression of recombinant enzymes has become a precondition for commercial applications. To achieve the consistency of the internal circumstances, lipase genes from *P*. *fluorescens* and *P*. *alcaligenes* were usually expressed in homological hosts, while their expression levels are fairly low. However, some former studies indicated that expression of them in *Escherichia coli* might provide another option to achieve high expression level^14–16^.

On the other hand, *Pichia pastoris* is a popular yeast species which is frequently applied to express recombinant proteins both in the fields of scientific researches and industries. Generally, foreign gene can be integrated into genome stably through single crossover which happens in the region of His4 or AOXl with the purpose of generating recombinants with Mut^+^ phenotype (among which methanol is commonly used). Additionally, it may also be used to substitute for the AOX1 locus between the end sequence of 5-AOX1 and 3-AOX1 so as to produce MutS phenotype (among which methanol is gradually used). So far, over 350 recombinant proteins from human, bacteria, yeasts and filamentous fungi have been successfully expressed in *P*. *pastoris* systems, with protein production of more than 10 g/L in some cases^17–24^. There also are a few reports on the expression of *Pseudomonas* lipases in yeast^18,25,26^.

Moreover, fossil diesel fuel is expected to be partly substituted by biodiesel which is produced via transesterification of oil with short-chain alcohol. To date, chemical methods have been the mainstream of producing commercial biodiesel, while the requirement of excessive energy and environmental pollution appear to be its major disadvantages. Compared with chemical methods, enzymatic catalyzed methods by lipases have many advantages, such as mild reaction conditions, simplified procedure, and little pollution. But the key problem of enzymatic catalyzed methods is that lipases are expensive and easy inactivation. Thus, an efficient conversion of biodiesel depends on lipase characteristics and its high productivity, which can effectively reduce production cost to a great extent^27^.

Therefore, in this research, a new lipase gene, Pflip1 from *P*. *fluorescens* Pf0–1 was successfully expressed in heterologous hosts of *E*. *coli* and *P*. *pastoris*, respectively. The recombinant lipases were characterized with reference to biodiesel preparation. Then, they were further immobilized on the magnetic nanoparticles and used for transesterification of soybean oil into biodiesel to assess potential feasibility.

## Results

### Cloning and overexpression of pflip1

**Figure 1:**
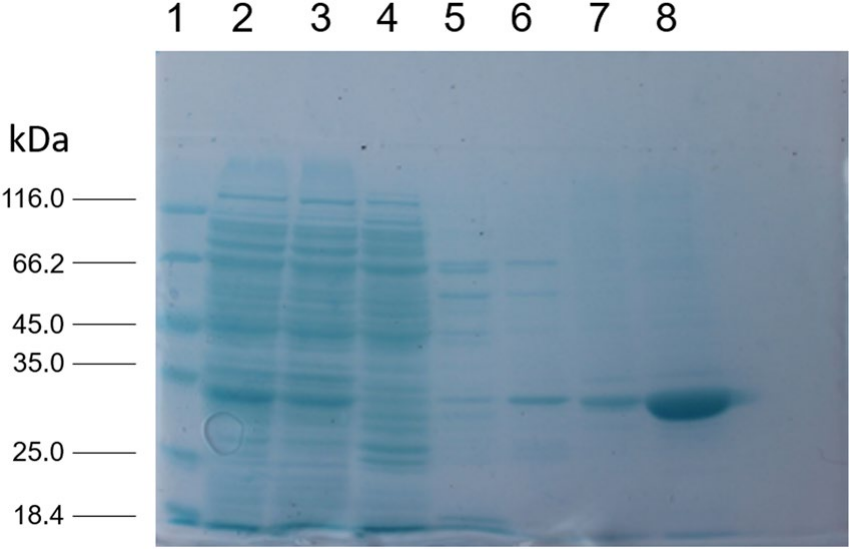
The result of SDS-PAGE gel showing the purified recombinant protein Pflip1a. Lane 1: Protein molecular weight marker (Fermentas, SM0431). Lane 2: supernatants of recombinant *E*. *coli* BL21 (DE3) cells lysates harbouring Pflip1a. Lane 3: flow-through buffer. Lane 4-Lane 8: washing buffer with an imidazole concentration gradient (30, 60, 100, 200, and 500 mM, respctly). The recombinant Pflip1a lipase is marked by an arrow in the figure.

The *Pflip1* gene (891 bp) was obtained by touchdown PCR using primers Pflip1F and Pflip1R with *P*. *fluorescens* Pf0–1 chromosomal DNA as template. Then, the gene *Pflip1* was respectively expressed in *E*. *coli* and in *P*. *pastoris* to obtain the recombinant proteins. In *E*. *coli*, under the influence of strong T7 promoter, the Pflip1 gene was inserted in pET28a and in translational fusion with His-tag. Upon the induction of IPTG, *E*. *coli* BL21(DE3) haboring the recombinant plasmid encoded a polypeptide of 296 amino acid, whose molecular mass was 32.1 kDa. After the break, the supernatant of the recombinant Pflip1a showed a moderate activity at 217 U/ml (438 U/mg) under its optimum conditions: pH 8.0; temperature 70 °C, with *p*-NP, *p*-nitrophenyl caprylate as substrates. Figure 1 showed the result of SDS-PAGE analysis. The molecular mass of the recombinant protein was ca 32 kDa, which coincides with the actual one.

**Figure 2:**
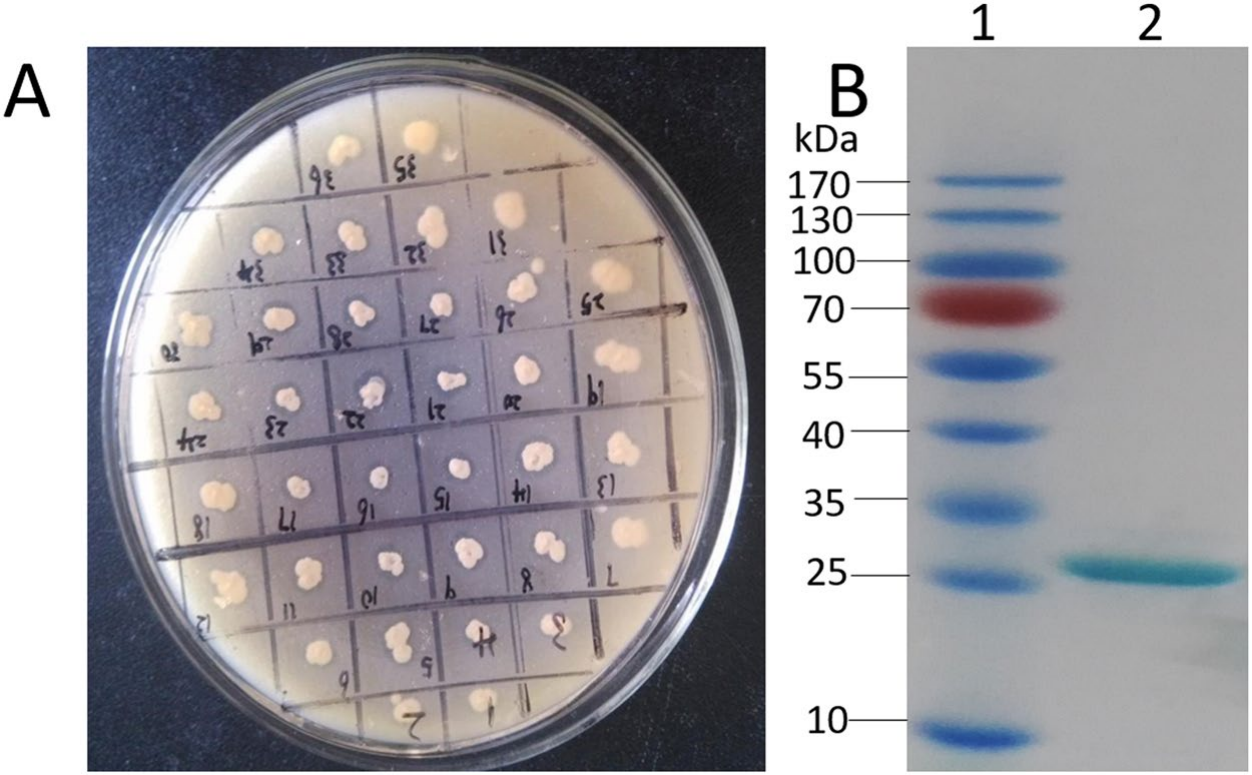
Enzyme activity identification and SDS-PAGE analysis of recombinant protein. (**A**) Colonies had some hydrolysis circles in the middle of the tributyrin plate, which indicated that recombinant *P. pastoris* cells were with enzyme activity. (**B**) The result of SDS-PAGE gel showing the purified recombinant protein Pflip1b.

Similarly, in *P*. *pastoris*, the Pflip1 gene was inserted in pPIC9K and in translational fusion with His-tag. Then, it was transformed into the *P*. *pastoris* strain KM71 cells. After the cells were spread in MD plate, a single clone was selected to grow in tributyrin plate, colonies with a clear hydrolysis holas were selected to grow in BMMY medium broth (Fig. 2A). The highest activity of the supernatant of fermentation in BMMY medium broth was measured to be 105 U/ml (336 U/mg). The purified recombinant protein, Pflip1b was analyzed via SDS-PAGE, and the result was shown in Fig. 2B.

### Molecular structure modeling

**Figure 3:**
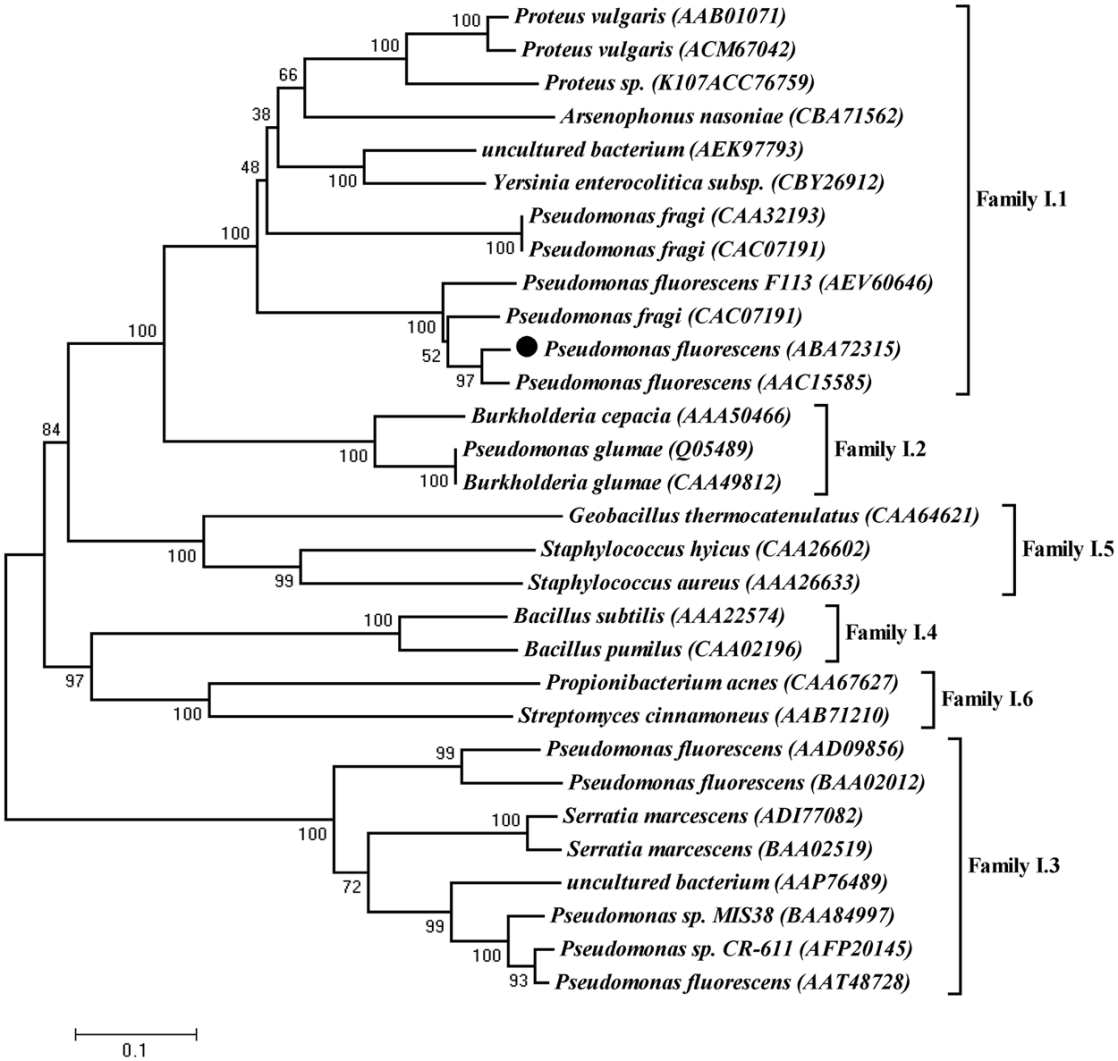
Phylogenetic tree of Pflip1 and other closely related lipolytic enzymes. The phylogenetic analysis was accomplished with the neighbor-joining method of MEGA 5.0. The values at nodes indicated the bootstrap percentage of 1,000 replicates, the lengths of the branches shown the relative divergence among the reference lipase amino acid sequences, and the scale bar indicated the amino acid substitutions per position. Database accession numbers were shown in brackets after each enzyme.

The Pflip1 gene encoded a 296 amino acid sequence with a theoretical molecular mass of 32.09 kDa. Further BLASTP analysis proved that Pflip1 shown high similarity to AAC15585, a lipase from *P*. *fluorescens* (identity: 92.6%); AEV60646, a triacylglycerol lipase from *P*. *fluorescens* F113 (identity: 82.1%); WP_015093259, an lipase from *Pseudomonas* sp. UW4 (identity: 85.5%); CAA32193, a unnamed protein product from *P*. *fragi* (identity: 43.9%); CAC07191, a lipase from *P*. *fragi* (identity: 47.1%), which indicates that Pflip1 is a bacterial I.1 subfamily lipase (Fig. 3). To classify Pflip1, a phylogenetic tree was built for the lipolytic enzymes which represent five different lipase subfamilies. As shown in Fig. 3, Pflip1 is really in subfamily I.1.

**Figure 4:**
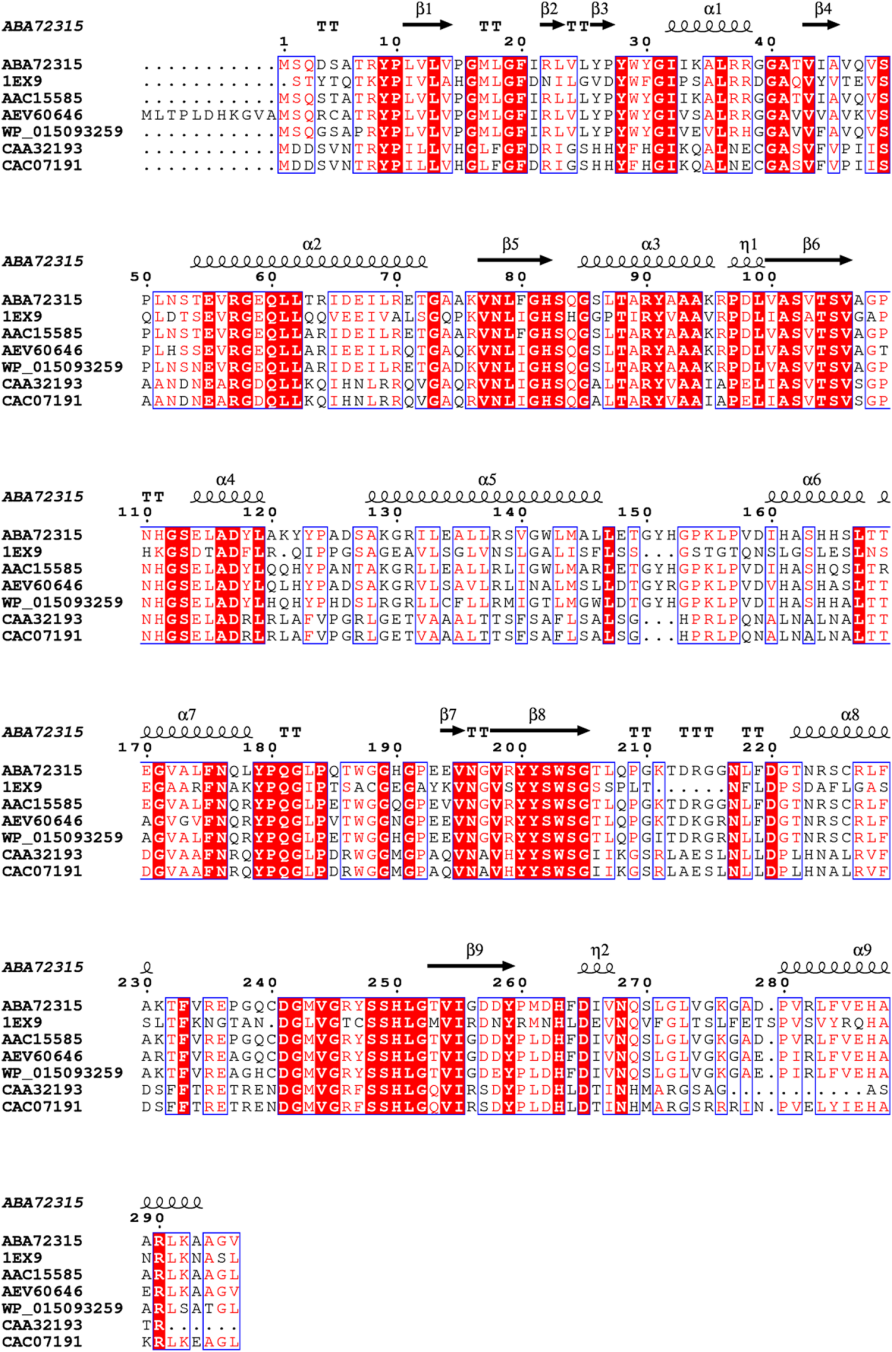
Multiple sequence alignment between Pflip1 and other closely related lipolytic enzymes: IEX9, a lipase from *Pseudomonas aeruginosa*; AAC15585, a lipase from *P*. *fluorescens*; AEV60646, a triacylglycerol lipase from *P*. *fluorescens* F113; WP_015093259, an lipase from *Pseudomonas sp*. UW4; CAA32193, a unnamed protein product from *P*. *fragi*; CAC07191, a lipase from *P*. *fragi*. Cluster 1.83 and ESpript 2.0 were used to analyze multiple sequence alignment. The alpha helix, beta sheet, random coil, and beta turn are identical to α, β, η, and T, respectively.

**Figure 5:**
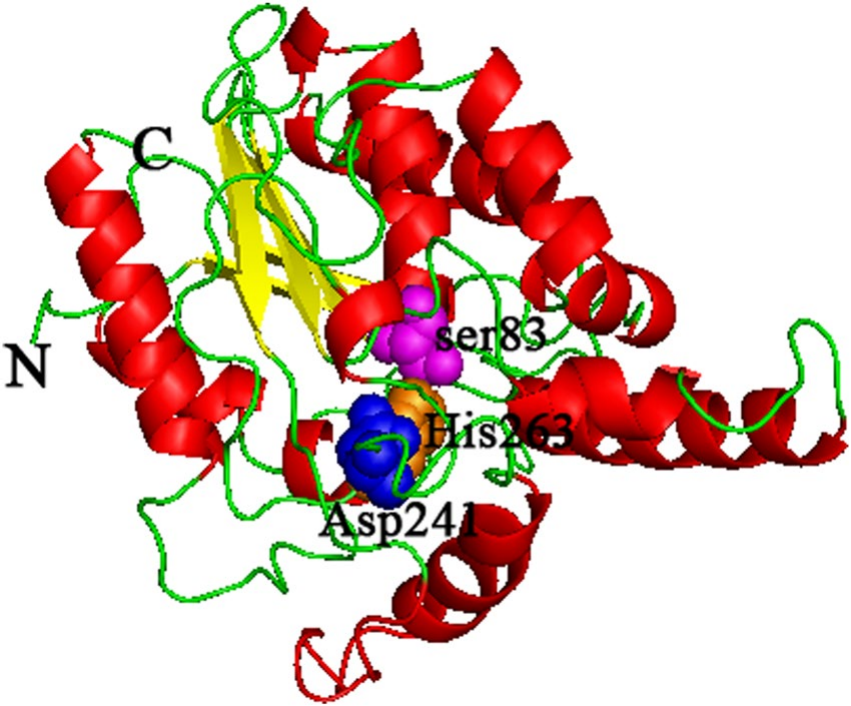
3D model of Pflip1. The α-helix, β-sheet, random coil and beta turn are shown in the cartoon in red, yellow and green, respectively. The catalytic triads (Ser83, Asp241 and His263) are shown as spheres in magenta, blue and orange, respectively. “N” and “C” denote the N and C termini, respectively.

Through searching in the RCSB PDB Protein Database, it was discovered that the crystal structure of the lipase (with accession number of IEX9) from *P*. *aeruginosa* had the largest identity of amino acid sequence (46.8%) with Pflip1. Therefore, it is deemed as the most appropriate template for homology modeling^28^. According to validation completed by online PROCHECK (http://nihserver.mbi. ucla.edu/SAVES/), the most preferred regions contains 86.2% of the residues in the modeled structure, and only 3 out of 296 amino acids are located in the disallowed regions, which indicates that the model is reliable. The 3D structure showed a variant of the α/β hydrolase fold, and Ser83, Asp241, and His263 as the catalytic active center. The first two β-strands and one α-helix (αE) are present compared with the “canonical” α/β hydrolase fold. The formation of a stabilizing intramolecular disulfide bridge derived from the absence of helix αE. The octahedrally-coordinated calcium ion is used to stabilize the His263-containing loop (Figs 4 and 5).

### Characterization of recombinant lipase Pflip1a

**Figure 6:**
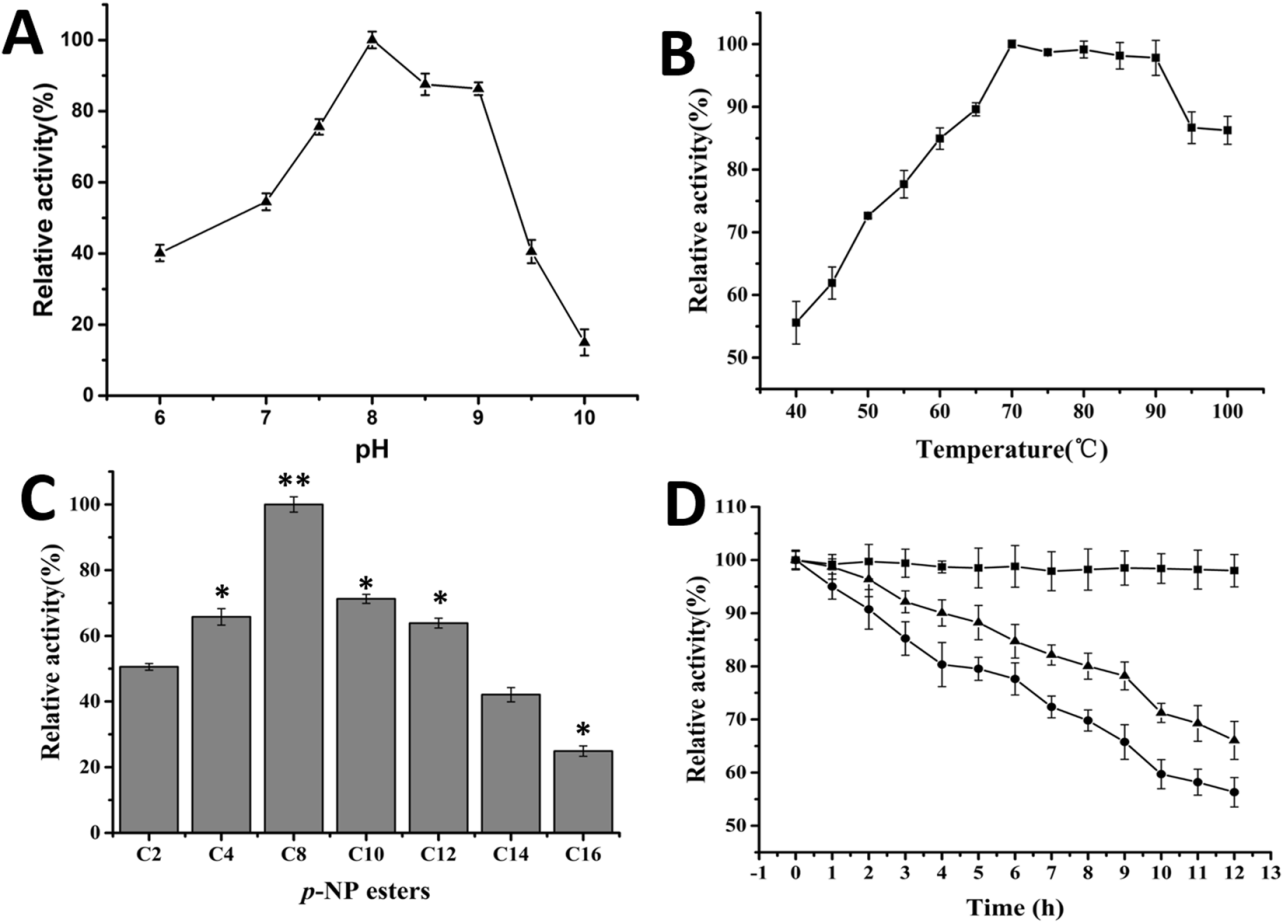
Characterization of Pflip1a. (**A**) Effect of pH on the activity of Pflip1a. The enzyme activity in Tris-HCl (50 mM, pH 8.0) was taken as 100%. (citrate-phosphate buffer (pH 5.0–6.5), Tris-HCl buffer (pH 7.0–8.5), Glycine-NaOH buffer (pH 9.0–10.0), and final concentration all were 50 mM). (**B)** Effect of temperature on the activity of Pflip1a. (40 °C–100 °C). (**C**) Effect of *p*-NP esters of different chain lengths on the activity of Pflip1a. (C2, C4, C8, C10, C12, C14, and C16). (**D**) Thermal stability of Pflip1a. The enzyme was incubated at 70 °C (■), 90 °C (▲), 80 °C (●).

The relative molecular mass of recombinant protein Pflip1a is ca 32 kDa, and Fig. 1 shows the SDS-PAGE analysis of Pflip1. The recombinant protein was active at a pH range from 5.0 to 10.0, and the optimum pH was 8.0 (Fig. 6A). The recombinant protein retained more than 75% of its maximal activity at a pH value range of 7.5 to 9.0. And the recombinant protein was active between 40 °C and 100 °C, especially between 70 °C and 90 °C, it retained at least 97.3% of its maximal activity at temperature range from 70 °C to 90 °C. Its optimal temperature was 70 °C (Fig. 6B).

The purified recombinant lipase could hydrolyze *p*-NP esters, it was active in *p*-NP esters of acyl fatty acid chain lengths ranging from C2 to C16, and showed the optimum activity in C8. Compared with C8, the relative activities of Pflip1a in C10 and C12 were 71.3% and 63.9%, respectively. In contrast, it has better activity towards the esters of short-chain and medium-chain fatty acids compared with those of long-chain fatty acids (Fig. 6C).

The thermostability of Pflip1a was examined in Tris-HCl (pH 8.0) at 70 °C, 80 °C, and 90 °C for 12 h (Fig. 6D). More than 70% of its maximal activity remained after incubation at 80 °C for 10 h, and about 70% of residual activity was retained after exposure at 90 °C for 7 h. The results indicate that Pflip1a has very good thermostability (Fig. 6D).

**Figure 7:**
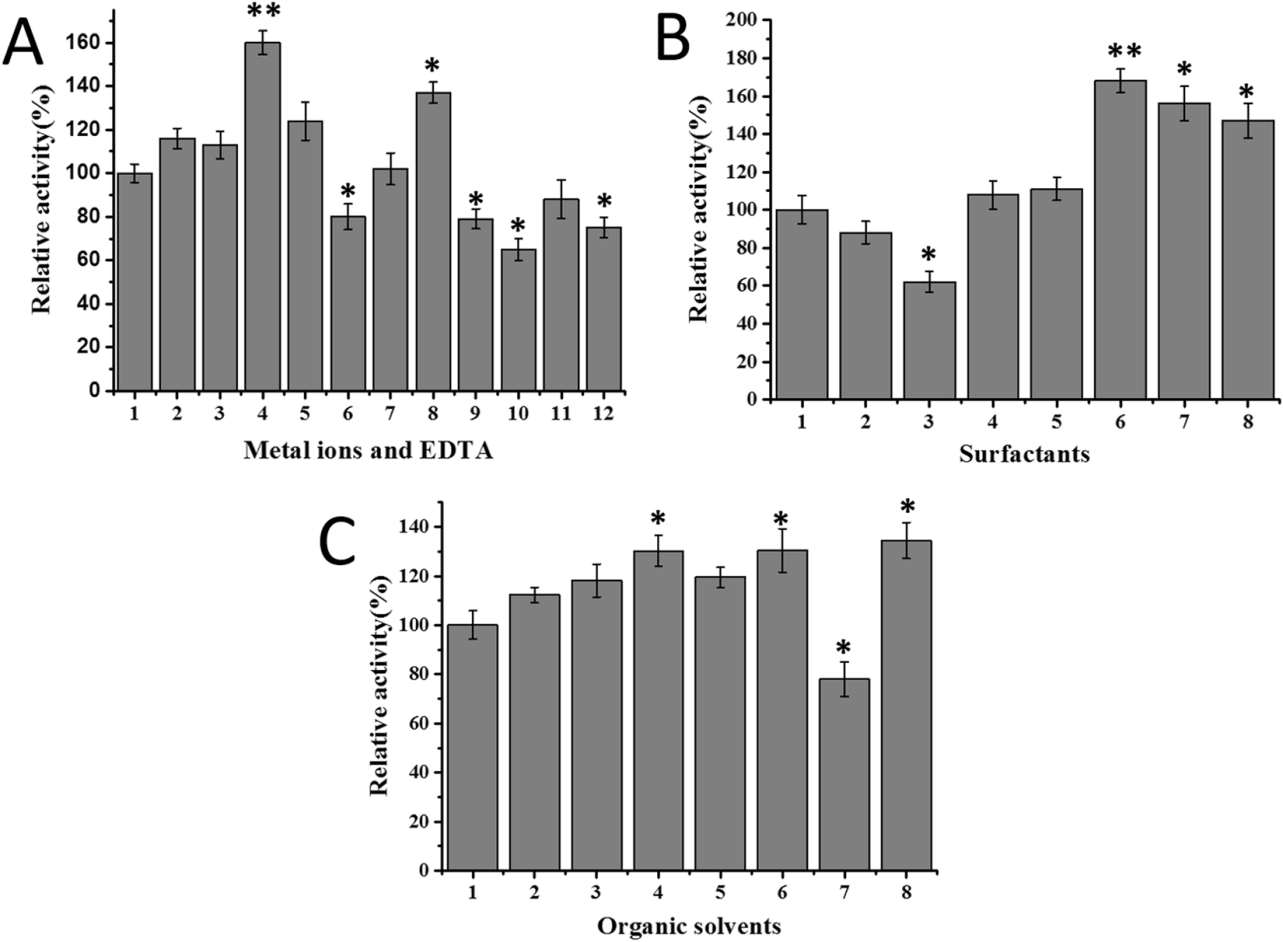
Effects of additives on Pflip1a activity. (**A**) Effects of metal ions and EDTA on the activity of Pflip1a. 1, control; 2, Na^+^; 3, K^+^; 4, Ca^2+^; 5, Mg^2+^; 6, Cu^2+^; 7, Mn^2+^; 8, Ba^2+^; 9, Zn^2+^; 10, Fe^3+^; 11, Al^3+^; 12, EDTA. (**B**) Effects of surfactants on the activity of Pflip1a. 1, control; 2, SDS; 3, CTAB; 4, NP-40; 5, Triton X-100; 6, Tween 20; 7, Tween 40; 8, Tween 80. (**C**) Effects of organic solvents on the activity of Pflip1a. 1, control; 2, methanol; 3, ethanol; 4, *tert*-butanol; 5, glycerol; 6, acetone; 7, chloroform; 8, *n*-hexane.

The effects of various metal ions and EDTA on the activities of Pflip1a were presented in Fig. 7A. The results showed that Ca^2+^, Mg^2+^, Ba^2+^ significantly enhanced its activity by 30.2%, 24.0% and 25.6%, respectively. On the contrary, Cu^2+^, Zn^2+^, Fe^3+^, Al^3+^ and EDTA markedly decreased its activity by 19.4%, 20.6%, 34.5%, 11.9% and 24.8%, whereas, Na^+^, K^+^, Mn^2+^ had no remarkable effect on its activity.

The effects of various surfactants on the activities of the Pflip1a were given in Fig. 7B. The results indicated that ionic surfactants SDS and CTAB remarkably inhibited its activity by 11.9% and 37.3%, whereas, non-ionic surfactants NP-40, Triton X-100, Tween 20, Tween 40, and Tween 80 significantly increased its activity by 8.4%, 11.3%, 31.3%, 29.0%, and 17.4%, respectively.

The activities of the Pflip1a in various organic solvents were also tested (Fig. 7C). It was only inhibited in the presence of chloroform (78.1%), whereas, other organic solvents stimulated its activity to some extent, and the maximum activity was exhibited in the presence of *n*-hexane (134.4%), followed by acetone (130.3%), *tert*-butanol (130.2%), ethanol (118.1%), glycerol (119.4%), and methanol (112.3%).

### Biodiesel preparation using the immobilized lipase Pflip1b and Pflip1a and their recyclability

**Figure 8:**
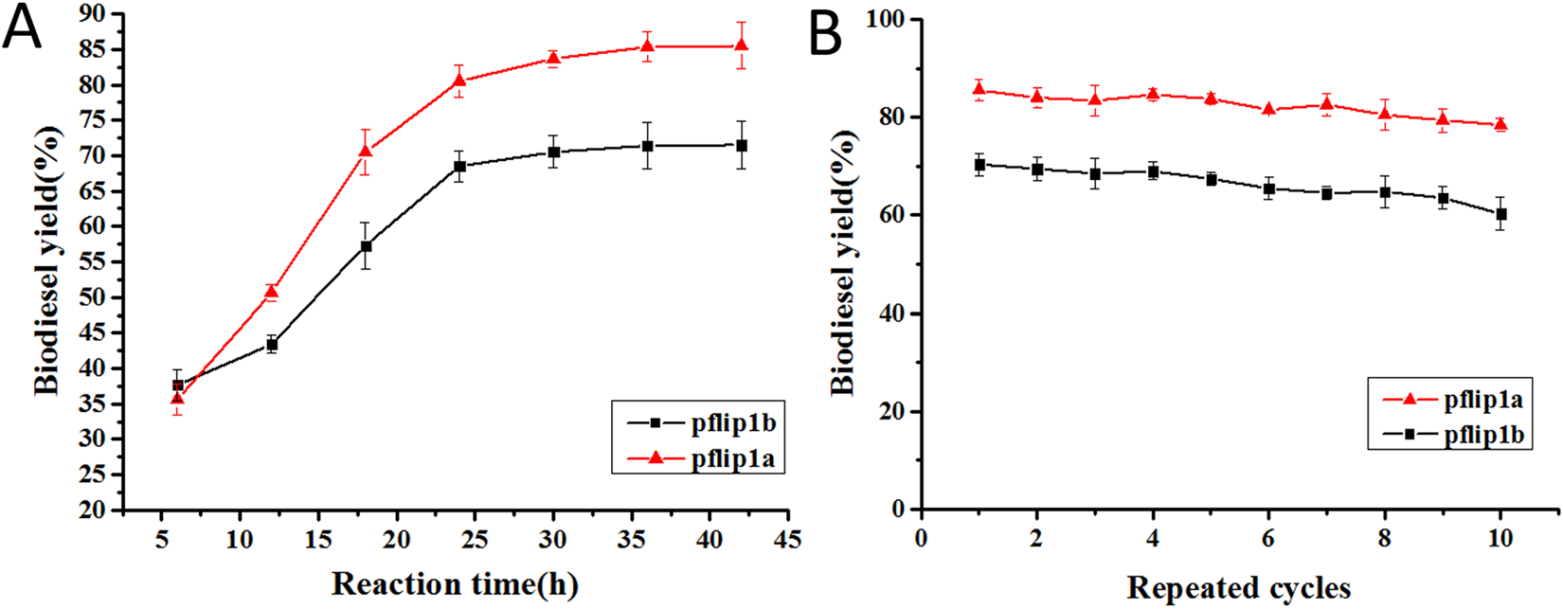
Conversion of soybean oil to biodiesel. (**A**) Detection of esterification reaction products by GC; (**B**) Reusability of the immobilized lipases Pflip1b and Pflip1a.

In the present study, immobilization treatment strategy was employed for lipases Pflip1b and Pflip1a to catalyze the transesterification of soybean oil into biodiesel. The two lipases were covalently attached on magnetic nano-particles. The reaction conditions were given in Section ‘Materials and Methods’. The immobilization efficiencies of immobilized Pflip1a and Pflip1b were 84.2% and 76.4%. The activity recoveries of immobilized Pflip1a and Pflip1b were 182.5% and 166.7%, respectively. The activities have been increased 1.8 and 1.7-fold for Pflip1a and Pflip1b after immobilization. The GC analysis (Fig. 8A) showed the biodiesel conversion for the immobilized lipases Pflip1b and Pflip1a. It can be seen that the substrate conversion rates after 24 h reaction at 70 °C were 68.5% and 80.5%, respectively. However, the conversion rates were significantly different between lipase Pflip1b and Pflip1a.

Reusability is one of the most important factors for the industrial applications of enzyme. The operational stability of the immobilized Pflip1b and Pflip1a were respectively presented in Fig. 8B. It can be seen from Fig. 8B that the two immobilized lipases showed high operational stability. The conversion rate of the two immobilized lipases had no obvious loss and still remained 70% and 82% of its original activity after running for 10 cycles in the solvent-free system.

## Discussion

*P*. *fluorescens* species was widely existed in the environment. These bacteria could be easily separated, so they were an excellent source of lipase gene resources. In this study, a new *P*. *fluorescens* lipase was first cloned and respectively overexpressed in *E*. *coli* and *P*. *pastoris*, the enzymatic property of Pflip1a was characterized, and both recombinant enzymes were further applied in biodiesel production. There were many lipase genes that had been cloned from genus *Pseudomonas*. Nevertheless, there were many unsolved problems in expression of the proteins encoded by these genes in *E*. *coli*. In addition, many recombinant proteins, including SIK W1 lipase isolated from *P*. *fluorescens* ^28^, LipM from *P*. *moraviensis* M9^29^, and LipR from *Pseudomonas* sp. R0–14^30^, form inclusion bodies because of incorrect folding or excessively rapid expression and accumulation of protein^31^. In this research, when Pflip1 of *P*. *fluorescens* Pf0–1 was transformed into *E*. *coli* BL21 (DE3) induced at low temperature, Pflip1a was overexpressed in a soluble and catalytically active form. This result indicates that the thermostable lipase genes may be expressed in psychrophilic and mesophilic organisms, and *E*. *coli* can slow down expression level of heterologous protein at low temperature, so it is expressed in soluble form. So far, many lipase genes from bacteria, yeasts and filamentous fungi have been functionally expressed. However, there were few reports on the expression of *Pseudomonas* lipases in yeast in comparison with effective expression of lipase genes in other organisms. Due to the differences of prokaryotic and eukaryotic expression systems, it is generally difficult for bacterial lipase to be expressed in active form in yeast. Nevertheless, in this study, we tried to express the bacterial a new lipase gene, *P*. *fluorescens* lipase gene in *P. pastrois* cells. The purified enzyme Pflipb was obtained. The catalytic activity of Pflip1b was much weaker than Pflip1a from *E*. *coli*, it shows that bacterial lipases are more suitable to express in prokaryotic expression system.

Among the members of I.1 lipase subfamily discussed above, Pflip1a showed its unique thermophilic property. The optimal hydrolyzing temperature for C8 was 70 °C, significantly higher than other homologous lipases. And high activity can be maintained continuously at high temperature of 70 °C. The lipase KB700A derived from *Pseudomonas* sp. displayed an optimal temperature of 35 °C^32^, and that of SIK W1 lipase came from *P*. *fluorescens* displayed a range of 45–55 °C^28^. This indicates that Pflip1a has great potential for various high temperature industrial applications.

It was reported that a majority of heavy metal ions deactivated lipases from *Pseudomonas* ^31^. But in this study, Pflip1a was enhanced by Ca^2+^, Mg^2+^, Ba^2+^, and other metal ions inhibited its activity. LipC12 from *Yersinia enterocolitica subsp. palearctica* Y11 had a preference for binding Ca^2+^, its maximum activity was achieved with addition of Ca^2+^, there may be a calcium ion hole for LipC12 dependent^33^. Similar to LipC12, the activity of Pflip1a was also heavily dependent on calcium ion. The activation by Ca^2+^ proved that the calcium-binding domain was important for the activity of Pflip1a. The fact that some divalent metal ions, including Ca^2+^, Mg^2+^ and Ba^2+^ could activate Pflip1a indicates that metal ions appear to be replaceable. Generally, non-ionic surfactants, such as Tween 20, Tween 40, Tween 60, Tween 80, and NP-40, and ionic surfactants, including SDS and CTAB, usually inhibited the activity of lipases^34^. But Pflip1a had good tolerance to these surfactants. Only SDS and CTAB inhibited its activity, other surfactants could promote. In particular, Tween 20 had a significant promoting effect on the improvement of enzyme activity. As known, lipases could be interfacial-activated due to the oil–water interface^27^. As a class of surfactants, Tween 20 may greatly promote the activation of lipase interface. The industrial applications of lipases could be broadened and enhanced greatly by using them in micro- or non-aqueous systems. Thus, high stability and activity of lipases in the presence of organic solvents were a prerequisite and desirable for biotransformation^31^. In view of this, the activities of Pflip1a in various organic solvents were also investigated. Pflip1a had an excellent tolerance to organic solvents, it was only inhibited in the presence of chloroform, and other organic solvents could stimulate its activity. These results show that Pflip1a has a potential for application in organic synthesis and transesterification.

The preparation of lipase-catalyzed biodiesel was generally recognized as an environmental-friendly technique. An efficient enzyme for biodiesel conversion is popularly welcomed. Its high productivity could effectively reduce the utilization cost of lipases to a great extent. Lipases originated from different biological sources usually have their own special characteristics. Some previous researches have proved that lipases from the same species of *P*. *fluorescens* exhibited different transesterification ability^35–37^. In our study, the biodiesel conversion rate of *P*. *fluorescens* Pflip1 reached 83.8% and that of Pflip1a is obviously higher than Pflip1b. The probable reason is attributable to the modifications of post translation in *P. pastoris* affecting the catalytic activity of the recombinant bacterial lipases. This to say, too much modification is not necessary for the proper folding of the lipase. Although most of the current used lipases are expressed in fungal systems, sometimes expression of bacterial lipase in prokaryotic system is more suitable.

## Materials and Methods

### Bacterial Strains, Plasmids and Chemicals

*P*. *fluorescens* Pf0–1 was preserved in our laboratory, *E*. *coli* TOP10 (Novagen, Darmstadt, Germany) and *E*. *coli* BL21(DE3) (Novagen, Darmstadt, Germany) were grown at 37 °C in LB medium or on agar plates. *P*. *pastoris* strain KM71 was grown in YPD broth (2% tryptone, 1% yeast extract, 2% dextrose) at 28 °C, or BMMY broth (1% yeast extract, 1.34% YNB, 4 × 10^−5^% biotin, 2% dextrose, 100 mM potassium buffer, 0.5% methanol) at 28 °C, or MD broth plates (2% dextrose, 4 × 10^−5^% biotin, 1.34% YNB and 2% agar) at 28 °C for screening yeast recombinants, or tributyrin agar plate (2% tryptone, 1% yeast extract, 1.34% YNB, 100 mM potassium buffer, 4 × 10^−5^% biotin, 1% glycerol at 16 °C, 1% tributyrin and 2% agar) at 28 °C. pMD19-T cloning vector (Takara, Otsu, Japan), pET-28a (Novagen, Darmstadt, Germany) and the plasmid pPIC9K (Invitrogen, Carlsbad, USA) were applied for constructing the expression plasmids. The antibiotics ampicillin (Ap; 100 μg/ml) and kanamycin (Km; 50 μg/ml) were used for screening in this study.

All strains were saved in 15% (v/v) glycerin solution and frozen at −20 °C and −80 °C. DNA sequencing has been completed by Invitrogen Biotechnology Company (Guangzhou, china). Taq polymerase, Restriction enzyme and T4 ligase were obtained from Takara (Otsu, Japan). Substrate *p*-nitrophenyl (*p*-NP) esters were purchased from Sigma-Aldrich (St.Louis, USA). And other chemicals of analytical grade were purchased from Sinopharm (Beijing, China). Primers used in the experiments are listed in Table 1.

**Table 1:** Primers used in this study.

Name	Sequence (5′-3′)	Annotation
Pflip1aF	GGAATTCCATATGATGTCGCAAGATTCGGCCACAC	*Nde*I(underlined)
Pflip1aR	CCCAAGCTTCTAGACCCCGGCGGCTTTCAAC	*Hind*III(underlined)
Pflip1bF	CGGAATTCATGTCGCAAGATTCGGCCACAC	*EcoR*I(underlined)
Pflip1bR	ATTTGCGGCCGCCTAGTGGTGATGATGGTGATGGACCCCGGCGGCTTTCAAC	NotI(underlined) (His) 6-tagged(wave lined)

### DNA manipulations

Genomic DNA of Pf0–1 was extracted by Bacteria Genomic DNA Kit (CWBIO, Beijing, China) for genome extraction. It was obtained via Touchdown PCR with degenerate primers Pflip1F and Pflip1R.

### Functional expression and purification of recombinant lipase Pflip1a in *E*. *coli*

After amplified with primers Pflip1aF and Pflip1aR, the PCR product was inserted into pET-28a by digested with *Nde* I and *Hind* III, such that the recombinant protein would have a carboxyl terminal of 6-his-tag. Then, it was transformed into the expression strain *E*. *coli* BL21(DE3) and grown overnight at 37 °C in a 5 ml LB medium. Later, the mixture was diluted with 1:100 into 1 L of LB broth containing ampicillin (100 μg/ml) and grown. After the optical density of the mixture at OD_600_ was between 0.4 and 0.5, the recombinant Pflip1a was induced with 0.1 mM IPTG at 16 °C for 20 h.

After centrifugation at 7,000 × g for 20 min at 4 °C and resuspension in lysis buffer (0.5 M NaCl, 20 mM Tris-HCl, pH 8.0), 6 L of culture was harvested and then disrupted by a One Shot Cell Disrupter (Constant Systems, Daventry, UK). Insoluble material was isolated by centrifugation at 12,000 × g for 20 min at 4 °C and supernatant was then passed through a 0.45 µm syringe-end filter before loading on His-Tag column. After that, the supernatants were transferred to a Ni-NTA affinity chromatography column (GE Healthcare, Pittsburgh, PA, USA). After the column balanced with lysis buffer, the recombinant protein was added into it and then eluted using washing buffer with an imidazole concentration gradient of 0, 30, 60, 100, 200, and 500 mM. Finally, 30 ml of the washing buffer containing the target recombinant enzymes was dialyzed in 20 mM Tris-HCl buffer (pH 8.0).

Then, SDS-PAGE was used to analyze the recombinant lipases. Unstained and pre-stained protein markers (Fermentas, SM0431 and SM0671 Waltham, USA) were used as references.

### Functional expression and purification of recombinant lipase Pflip1b in *P*. *pastoris*

After amplified with primers Pflip1bF and Pflip1bR, the PCR product was cloned into pPIC9K by digested with *EcoR* I and *Not* I and ligated. It had a carboxyl terminal of 6-his-tag. Then, it was transformed into *P*. *pastoris* strain KM71 cells. Transformants grew on MD plates at 28 °C for 2–3 days. Subsequently, 25 colonies with large hydrolyzing halos were picked up and grown overnight in the YPD at 28 °C. These colonies was diluted with 1:100 into 1 L of BMMY broth and grown for 7 days.

After centrifuged at 7,000 × g for 20 min at 4 °C and passed through a 0.45 µm syringe in a concentration tube, 6 L of the supernatants was concentrated and collected. Then, the concentrated solution (concentrated for 20 times) was transferred to a Ni-NTA affinity chromatography column. The other purification procedure of the recombinant protein is similar to the above-mentioned Pflip1a. The analysis method is similar to the above stated.

### Biochemical characterization of Pflip1a

The optimal substrate was examined using a standard assay except for different *p*-nitrophenyl esters as substrates, they were *p*-nitrophenyl (*p*-NP) acetate (C2), butyrate (C4), *p*-NP hexanoate (C6), *p*-NP caprylate (C8), *p*-NP decanoate (C10), laurate (C12), *p*-NP myristate (C14), and *p*-NP palmitate (C16), respectively. The optimum pH was analyzed through measuring the enzyme activity at 70 °C with C8 as substrate and in different buffers with pH ranging from 5.0 to 10.0 (citrate-phosphate buffer,pH 5.0–6.5; Tris-HCl buffer, pH 7.0–8.5; glycine-NaOH buffer, pH 9.0–10.0). The temperature effect was tested using a standard assay except for varied temperatures (40 °C-100 °C). In a standard assay, the total reaction system of 1 ml contains 940 μl of Tris–HCl buffer (50 mM, pH 8.0), 10 μl of *p*-NP ester (100 mM, soluble in acetonitrile), 40 μl of ethanol and 10 μl of the diluted enzyme solution. The blank contained the same components without addition of enzyme solution^38^. Unless otherwise described, lipase activity was measured in this standard assay at 70 °C for 10 min. All experiments were performed in triplicates. The mixtures were centrifuged, and the absorbance of the resulting supernatants was measured at 410 nm by use of UV-1600 spectrophotometer (MAPADA, Shanghai, China)^39,40^. One unit of lipase activity (U) was defined as the amount of enzyme that released 1 μmol *p*-NP per minute under the assay conditions.

The effects of metal ions (Na^+^, K^+^, Mn^2+^, Ca^2+^, Mg^2+^, Ba^2+^, Cu^2+^, Zn^2+^, Fe^3+^, and Al^3+^) and the chelating agent EDTA on enzyme activity were determined by adding 10 mM of each into the reaction system and then followed by a lipase activity assay. The influences of detergents (SDS, CTAB, Tween-20, NP-40, Triton X-100, Tween 20, Tween 40, and Tween 80) on enzyme activity were investigated by measuring the retaining activities through addition of 1% v/v of various detergents into the reaction system. After 1 h treatment, the residual activities were again measured in the standard assay conditions. For the organic solvent tolerance of the purified enzymes, aliquots of the recombinant enzymes were incubated in 30% of organic solvents such as chloroform, methanol, acetone, ethanol, tert-butanol, glycerol and n-hexane. After 2 h treatment, the residual activities were measured in the standard assay conditions as stated above.

### Sequence analysis

The *Pflip1* accession numbers of nucleotide sequence and amino acid sequence in the NCBI (http://www.ncbi.nlm.nih.gov/) were Pfl01_0571 and ABA72315.1, respectively.

The BlastP program of NCBI (http://blast.ncbi.nlm.nih.gov/) were applied to search for protein sequence similarity. ClustalW (version 1.83) and ESPript 2.2 (http://espript.ibcp.fr/ESPript/ESPript/) were employed to analyze multiple sequence alignments^41^. Phylogenetic tree analysis was accomplished with the neighbor-joining method of MEGA 5.0^42^. A bootstrap analysis with 1000 replicates was applied to estimate the reliability of the tree. The ExPASy proteomics server (http://us.expasy.org/tools/protparam.html) was adopted for the analysis of protein physicochemical parameters (ProtParam tool).

### Immobilization of the two recombinant lipases on magnetic nanoparticles and enzymatic properties assay

The purified recombinant lipases were separately immobilized on magnetic nanoparticles (self-made in our lab) in aqueous phase as follows^27^: 0.5 ml Pflip1a lipase solution (0.19 mg-protein/ml) and 0.5 ml Pflip1b lipase solution (0.17 mg-protein/ml) were respectively added in 4 ml 0.05 M Tris-HCl buffer (pH 8.0) containing 0.5 ml magnetic nano-particles activated by glutaraldehyde solution (i.e. MNP-G). The mixture was shaken at 30 °C for 2 h. Subsequently, the immobilized lipases were easily separated with a magnet and washed for several times with buffer. The enzymatic activities of the free lipase and the immobilized lipase were assayed by adopting the method obtained previously^27^. A brief description is^29^: 1 ml reaction system contained 940 µl of Tris-HCl buffer (50 mM, pH 8.0), 10 µl of *p*-NP caprylate (C8, 100 mM), 40 µl of ethanol, and a certain amount of the free or immobilized lipase. The mixture was pre-incubated at 70 °C for 5 min before the reaction, and then incubated at 70 °C for 10 min. After doing so, 2 ml of acetone was added to quench the reaction. Then, the absorbance of *p*-nitrophenol released was recorded at OD_410_. A unit is defined as the amount of enzyme liberating 1 µmol of fatty acid per minute. Protein concentrations were measured by Bradford assay^43^. The blank contained all the components except for the dilution of the enzyme solution. All experiments were performed for three times.

### Transesterification of soybean oil into biodiesel and recyclability assay

The total reaction system containing 2.19 g soybean oil, 10 wt % immobilized enzyme, 5 wt % buffer (K_2_HPO_4_-KH_2_PO_4_, 0.1 mol/L, pH = 8.0) was conducted in a 50 mL shaking flask at 60 °C with a shaking speed of 200 rpm for 42 h^27,44,45^. The molar ratio of oil/ethanol was 1:4 and ethanol was added in three steps at the same interval of 6 h. Unless otherwise specified, all the dosage percentages were calculated on the basis of oil weight. The recyclability of the two immobilized lipases was further studied. By the end of the reaction, the immobilized lipases were separated from the mixtures with a magnet, washed with buffer, then under the same conditions reused for the new batch and calculated the biodiesel yield of each batch. The fatty acid alkyl esters (biodiesel) were measured by a GC-9790 gas chromatograph (Agilent HP-INNOWAX capillary column 30 m × 0.25 mm × 0.25 μm, J&W Scientific, Folsom, CA), and the operating conditions were subject to those described by Yan *et al*.^46^.

### Ethical approval

This article does not contain any studies with human participants or animals performed by any of the authors.

## Electronic supplementary material

Dataset 1

Dateset 2
