# Supplementary material for: Heterologous expression and characterization of a new lipase from Pseudomonas fluorescens Pf0–1 and used for biodiesel production
Source: Sci Rep. 2017 Nov 16;7:15711. doi: 10.1038/s41598-017-16036-7 (PMC5691200; doi:10.1038/s41598-017-16036-7)
Supplement: Supplementary file 1 — Dataset 1 [file 41598_2017_16036_MOESM1_ESM.docx]

**Heterologous expression and characterization of a new lipase from *Pseudomonas fluorescens* Pf0-1 and used for biodiesel production**

Wu Liu, Menggang Li, Yunjun Yan^*^

**FIG 3 Phylogenetic tree of Pflip1 and other closely related lipolytic enzymes**

>Pseudomonas fluorescens(ABA72315)

MSQDSATRYPLVLVPGMLGFIRLVLYPYWYGIIKALRRGGATVIAVQVSPLNSTEVRGEQLLTRIDEILRETGAAKVNLFGHSQGSLTARYAAAKRPDLVASVTSVAGPNHGSELADYLAKYYPADSAKGRILEALLRSVGWLMALLETGYHGPKLPVDIHASHHSLTTEGVALFNQLYPQGLPQTWGGHGPEEVNGVRYYSWSGTLQPGKTDRGGNLFDGTNRSCRLFAKTFVREPGQCDGMVGRYSSHLGTVIGDDYPMDHFDIVNQSLGLVGKGADPVRLFVEHAARLKAAGV

>Pseudomonas fluorescens(AAC15585)

MSQSTATRYPLVLVPGMLGFIRLLLYPYWYGIIKALRRGGATVIAVQVSPLNSTEVRGEQLLARIDEILRETGAARVNLFGHSQGSLTARYAAAKRPDLVASVTSVAGPNHGSELADYLQQHYPANTAKGRLLEALLRLIGWLMARLETGYHGPKLPVDIHASHQSLTREGVALFNQRYPQGLPETWGGQGPEVVNGVRYYSWSGTLQPGKTDRGGNLFDGTNRSCRLFAKTFVREPGQCDGMVGRYSSHLGTVIGDDYPLDHFDIVNQSLGLVGKGADPVRLFVEHAARLKAAGL

>Pseudomonas fluorescens F113(AEV60646)

MLTPLDHKGVAMSQRCATRYPLVLVPGMLGFIRLVLYPYWYGIVSALRRGGAVVVAVKVSPLHSSEVRGEQLLARIEEILRQTGAQKVNLIGHSQGSLTARYAAAKRPDLVASVTSVAGTNHGSELADYLQLHYPADSAKGRVLSAVLRLINALMSLLDTGYRGPKLPVDVHASHASLTTAGVGVFNQRYPQGLPVTWGGNGPEEVNGVRYYSWSGTLQPGKTDKGRNLFDGTNRSCRLFARTFVREAGQCDGMVGRYSSHLGTVIGDDYPLDHFDIVNQSLGLVGKGAEPIRLFVEHAERLKAAGV

>Pseudomonas sp. UW4(WP_015093259)

MSQGSAPRYPLVLVPGMLGFIRLVLYPYWYGIVEVLRHGGAVVFAVQVSPLNSNEVRGEQLLARIDEILRETGADKVNLIGHSQGSLTARYAAAKRPDLVASVTSVAGPNHGSELADYLHQHYPHDSLRGRLLCFLLRMIGTLMGWLDTGYHGPKLPVDIHASHHALTTAGVALFNQRYPQGLPETWGGHGPEEVNGVRYYSWSGTLQPGITDRGRNLLDGTNRSCRLFAKTFVREAGHCDGMVGRYSSHLGTVIGDEYPLDHFDIVNQSLGLVGKGAEPIRLFVEHAARLSATGL

>Pseudomonas fragi(CAA32193) I.1

MDDSVNTRYPILLVHGLFGFDRIGSHHYFHGIKQALNECGASVFVPIISAANDNEARGDQLLKQIHNLRRQVGAQRVNLIGHSQGALTARYVAAIAPELIASVTSVSGPNHGSELADRLRLAFVPGRLGETVAAALTTSFSAFLSALSGHPRLPQNALNALNALTTDGVAAFNRQYPQGLPDRWGGMGPAQVNAVHYYSWSGIIKGSRLAESLNLLDPLHNALRVFDSFFTRETRENDGMVGRFSSHLGQVIRSDYPLDHLDTINHMARGSAGASTR

>Proteus vulgaris（AAB01071） I.1

MENMSTTYPIVLVHGLSGFDDIVGYPYFYGIRDALEKDGHKVFTASLSAFNSNEVRGEQLWEFVQKVLKETKAKKVNLIGHSQGPLACRYVAAKHAKNIASVTSVNGVNHGSEIADLVRRIMRKDSVPEYIADAVMKAIGTIISTFSGNRGNPQDAIAALEALTTENVMEFNKKYPQGLPAIRGGEGKEVVNGVHYYSFGSYIQGLIAGEKGNLLDPTHAAMRVLSAFFTERENDGLVGRTSMRLGKLIKDDYAEDHLDMVNQVAGLVGPGEDIVAIYTNHANFLASKKL

>Proteus vulgaris（ACM67042） I.1

MSTTYPIVLVHGLSGFDNVVGYPYFYGIADALEKDGHKVFTASLSAFNANEVRGEQLWGFVQKVLKETKAKKVNLIGHSQGPLACRYVAAKHAKNIASVTSINGVNHGSEIADLVRRIVRKDSVPEYIADAVMKAIGTIISTFSGHRSNPQDAVAALEALTTENVTEFNKKYPQGLPAIRGGEGKEVVNGVYYYSFGSYIQGLIVGEKGNLLDPTHAAMRVLSAFFTEHENDGLVGRTSMRLGKLIKDDYAEDHLDMVNQVAGLVGRGEDIVAIYTNHANFLASKKL

>Proteus sp. K107（ACC76759） I.1

MSTKYPIVLVHGLAGFNEIVGFPYFYGIADALRQDGHQVFTASLSAFNSNEVRGKQLWQFVQTLLQETQAKKVNFIGHSQGPLACRYVAANYPDSVASVTSINGVNHGSEIADLYRRIMRKDSIPEYIVEKVLNAFGTIISTFSGHRGDPQDAIAALESLTTEQVTEFNNKYPQALPKTPGGEGDEIVNGVHYYCFGSYIQGLIAGEKGNLLDPTHAAMRVLNTFFTEKQNDGLVGRSSMRLGKLIKDDYAQDHIDMVNQVAGLVGYNEDIVAIYTQHAKYLASKQL

>uncultured bacterium（AEK97793） I.1

MSASSLKFPIVLVHGLLGFDKIGGIYPYFYGIKEALEKAGAKVYIATLSALNSNELRGEQLLEFVRKVQA

ETGAAKVNLIGHSQGPLACRYVAATHPELIASVTSVNGVNHGSEVADLVRLALTPGRLPESIANAAMSAFGQLLSALAGSPRLPQSGIEALEALTSEGVAAFNNKYPQGLPAEWGGEGKELVNGVYYYSWSGVIDYNPLHQGANNLDPLHVAMLAFSILFTNERFQNDGLVGRYSSHLGKVIGSDYSMDHVDAINQLAGVVANNTDPVQLFVEHVARLKSKGL

>Yersinia enterocolitica subsp. palearctica Y11（CBY26912） I.1

MSTNSTLKYPVVLVHGLLGFDKIAGVYPYFYGVEEPLKKAGAQVFVATISATNSNEVRGEQLLKFVKEVMAKTGAKKVNLIGHSQGPLACRYVAATHPELIASVTSVNGVNHGSEIADLVRSALVPGSLPEHIVNTIMSAFGVFVSLLSGKPFLPQDFMESIDALTTENVAKFNTKYPQGLPETWGGEGKEFDNGVYYYSWGGVLGYNPLIEGLNNLDPLHHSLVALSLLFTKERNQNDGLVGRYSMHLGKVIRSDYQLDHVDAINQTAGMVSKDIDPVQLFVNQIELLKSKGL

>Arsenophonus nasoniae（CBA71562） I.1

MPTKYPIILVHGLFGFDKIAGYPCFFAIENRLKKQGYNVFSPILSGVNSNEVNGEQLWLYIEELKRNIGC

EKVNLIAHSQGALFARYVAANYSASIASVTSMNGVNHGSEIADLVRKVLIPGKLTETVVVTAAEIFFTFI

SLISSGTLKPQDGVEALNSLTTEVVNEFNKKYPQGLPKEWRGEGDEVVNGVHYYSFGSFIKKSLINSGINAFDVSHTSLLQLSSFFSREKENDGLVGRYSMRLGKLICDDYDMDHLDIVNQIAGVVSNQYDIPSIYVNHAQMLAKKGL

>Pseudomonas fragi（CAC07191） I.1

MDDSVNTRYPILLVHGLFGFDRIGSHHYFHGIKQALNECGASVFVPIISAANDNEARGDQLLKQIHNLRRQVGAQRVNLIGHSQGALTARYVAAIAPELIASVTSVSGPNHGSELADRLRLAFVPGRLGETVAAALTTSFSAFLSALSGHPRLPQNALNALNALTTDGVAAFNRQYPQGLPDRWGGMGPAQVNAVHYYSWSGIIKGSRLAESLNLLDPLHNALRVFDSFFTRETRENDGMVGRFSSHLGQVIRSDYPLDHLDTINHMARGSRRRINPVELYIEHAKRLKEAGL

>Burkholderia cepacia(AAA50466) I.2

MARTMRSRVVAGAVACAMSIAPFAGTTAVMTLATTHAAMAATAPAAGYAATRYPIILVHGLSGTDKYAGVLEYWYGIQEDLQQNGATVYVANLSGFQSDDGPNGRGEQLLAYVKTVLAATGATKVNLVGHSQGGLSSRYVAAVAPDLVASVTTIGTPHRGSEFADFVQDVLAYDPTGLSSSVIAAFVNVFGILTSSSHNTNQDALAALQTLTTARAATYNQNYPSAGLGAPGSCQTGAPTETVGGNTHLLYSWAGTAIQPTLSVFGVTGATDTSTLPLVDPANVLDLSTLALFGTGTVMINRGSGQNDGLVSKCSALYGKVLSTSYKWNHLDEINQLLGVRGAYAEDPVAVIRTHANRLKLAGV

>Pseudomonas glumae(Q05489) I.2

MVRSMRSRVAARAVAWALAVMPLAGAAGLTMAASPAAVAADTYAATRYPVILVHGLAGTDKFANVVDYWYGIQSDLQSHGAKVYVANLSGFQSDDGPNGRGEQLLAYVKQVLAATGATKVNLIGHSQGGLTSRYVAAVAPQLVASVTTIGTPHRGSEFADFVQDVLKTDPTGLSSTVIAAFVNVFGTLVSSSHNTDQDALAALRTLTTAQTATYNRNFPSAGLGAPGSCQTGAATETVGGSQHLLYSWGGTAIQPTSTVLGVTGATDTSTGTLDVANVTDPSTLALLATGAVMINRASGQNDGLVSRCSSLFGQVISTSYHWNHLDEINQLLGVRGANAEDPVAVIRTHVNRLKLQGV

>Burkholderia glumae(CAA49812) I.2

MVRSMRSRVAARAVAWALAVMPLAGAAGLTMAASPAAVAADTYAATRYPVILVHGLAGTDKFANVVDYWYGIQSDLQSHGAKVYVANLSGFQSDDGPNGRGEQLLAYVKQVLAATGATKVNLIGHSQGGLTSRYVAAVAPQLVASVTTIGTPHRGSEFADFVQDVLKTDPTGLSSTVIAAFVNVFGTLVSSSHNTDQDALAALRTLTTAQTATYNRNFPSAGLGAPGSCQTGAATETVGGSQHLLYSWGGTAIQPTSTVLGVTGATDTSTGTLDVANVTDPSTLALLATGAVMINRASGQNDGLVSRCSSLFGQVISTSYHWNHLDEINQLLGVRGANAEDPVAVIRTHVNRLKLQGV

>Pseudomonas fluorescens(AAD09856) I.3

MGVFDYKNLGTEASKTLFADATAITLYTYHNLDNGFAVGYQQHGLGLGLPATLVGALLGSTDSQGVIPGIPWNPDSEKAALDAVHAAGWTPISASALGYGGKVDARGTFFGEKAGYTTAQAEVLGKYDDAGKLLEIGIGFRGTSGPRESLITDSIGDLVSDLLAALGPKDYAKNYAGEAFGGLLKTVADYAGAHGLSGKDVLVSGHSLGGLAVNSMADLSTSKWAGFYKDANYLAYASPTQSAGDKVLNIGYENDPVFRALDGSTFNLSSLGVHDKAHESTTDNIVSFNDHYASTLWNVLPFSIANLSTWVSHLPSAYGDGMTRVLESGFYEQMTRDSTIIVANLSDPARANTWVQDLNRNAEPHTGNTFIIGSDGNDLIQGGKGADFIEGGKGNDTIRDNSGHNTFLFSGHFGQDRIIGYQPTDRLVFQGADGSTDLRDHAKAVGADTVLSFGADSVTLVGVGLGGLWSEGVLIS

>Pseudomonas fluorescens(BAA02012) I.3

MGVFDYKNLGTEASKTLFADATAITLYTYHNLDNGFAVGYQQHGLGLGCRHTGRGVARQHRLPGSDPPAFPGILTRKRPPWTRCTQPVGRQSSASALGYGGKVDARGTFFGEKAGYTTAQAEVLGKYDDAGKLLEIGIGFRGTSGPRESLITTPCRSGQRPARRAGPQGLCEKLCRRTFGGLLKTVADYAGAHGLSGKDVLVSGHSLGGLAVNSMADLSTSKWAGFYKDANYLAYASPTQSAGDKVLNIGYENDPVFRALDGSTFNLSSLGVHDKAHESTTDNIVSFNDHYASTLWNVLPFSIANLSTWVSHLPSAYGDGMTRVLESGFYEQMTRDSTIILCPTWSDPARANTWVQDLNRNAEPHTGNTFIIGSDGNDLIQGGKGADFIEGGKGNDTIRDNSGHNTFLFSGHFGQDRIIGYQPTGWCSRAPTAAPTCATTRRPWGPIRC

>Pseudomonas sp. MIS38(BAA84997) I.3

MGVYDYKNFGTADSKALFSDAMAITLYSYHNLDNGFAAGYQHNGFGLGLPATLVTALLGGTDSQGVIPGIPWNPDSEKLALDAVKKAGWTPITASQLGYDGKTDARGTFFGEKAGYTTAQVEILGKYDAQGHLTEIGIAFRGTSGPRENLILDSIGDVINDLLAAFGPKDYAKNYVGEAFGNLLNDVVAFAKANGLSGKDVLVSGHSLGGLAVNSMADLSGGKWGGFFADSNYIAYASPTQSSTDKVLNVGYENDPVFRALDGSTFTGASVGVHDAPKESATDNIVSFNDHYASTAWNLLPFSILNIPTWISHLPTAYGDGMNRIIESKFYDLTSKDSTIIVANLSDPARANTWVQDLNRNAETHKGSTFIIGSDSNDLIQGGSGNDYLEGRAGNDTFRDGGGYNVILGGAGNNTLDLQKSVNTFDFANDGAGNLYVRDANGGISITRDIGSIVTKEPGFLWGLFKDDVTHSVTASGLKVGSNVTQYDASVKGTNGADTLKAHAGGDWLFGLDGNDHLIGGVGNDVFVGGAGNDLMESGGGADTFLFNGAFGQDRVVGFTSNDKLVFLGVQGVLPNDDFRAHASMVGQDTVLKFGGDSVTLVGVALNSLSADGIVIA

>Serratia marcescens(ADI77082) I.3

MGIFSYKDLDENASKALFSDALAISTYAYHNIGNGFDEGYHQTGFGLGLPLTLVTALIGSTQSQGGLPGLPWNPDSEQAAQEAVNNAGWSVISAAQLGYAGKTDARGTYYGETAGYTTAQAEVLGKYDSEGNLTAIGISFRGTSGPRESLIGDTIGDVINDLLAGFGPKGYADGYTLKAFGNLLGDAAKFAQAHGLSGEDVVVSGHSLGGLAVNSMAAQSDASWSGFYAQSNYVAFASPTQYETGGKVLNIGYENDPVFRALDGTTLTGTSLGVHDAPHTSATNNIVNFNDHYASDAWNLLPFSILNIPTWLSHLPFFYQDGLMRVLNSEFYSLTDKDSTIIVSNLSNITRGNTWVEDLNRNAETHSGPTFIIGSDGNDLIKGGKGNDYLEGRDGDDIFRDAGGYNLIAGGKGHNIFDTQQALKNTEVAYDGNTLYLRDAKGGITLADDISTLRSKETSWLIFNKEVDHQVTAAGLKSDSGLKAYAAAATGGDGDDVLQARSHDAWLFGNAGNDTLIGHAGGNLTFVGGSGDDVLKGVGNGNTFLFSGDFGRDQLYGFNASDKLVFIGTEGASGNIRDYATQQNDDLVLAFGHSQVTLIGVSLDHFSTDQVVLA

>Serratia marcescens(BAA02519) I.3

MGIFSYKDLDENASKALFSDALAISTYAYHNIDNGFDEGYHQTGFGLGLPLTLITALIGSTQSQGGLPGLPWNPDSEQAAQDAVNNAGWSVIDAAQLGYAGKTDARGTYYGETAGYTTAQAEVLGKYDSEGNLTAIGISFRGTSGPRESLIGDTIGDVINDLLAGFGPKAMRRYTLKAFGNLLGDVAKFAQAHGLSGEDVVISGHSLGGLAVNSMAAQSDATWGGFYAQSNYVAFASPTQYEAGGKVINIGYENDPVFRALDGTSLTLPSLGVHDAPHTSATNNIVNFNDHYASDAWNLLPFSILNIPTWLSHLPFFYQDGLMRVLNSEFYSLTDKDSTIIVSNLSNVTRGSTWVEDLNRNAETHSGPTFIIGSDGNDLIKGGKGNDYLEGRDGDDIFRDAGGYNLIAGGKGHNIFDTQQALKNTEVAYDGNTLYLRDAKGGITLADDISTLRSKETSWLIFSKEVDHQVTAAGLKSDSGLKAYAAATTGGDGDDVLQARSHDAWLFGNAGNDTLIGHAGGNLTFVGGSGDDILKGVGNGNTFLFSGDFGRDQLYGFNATDKLVFIGTEGASGNIRDYATQQNDDLVLAFGHSQVTLIGVSLDHFNPDQVVLA

>uncultured bacterium（AAP76489） I.3

MGVYDYKNFSTAESKALFTDAMAITLYSYHNLDNGFATGYQDNGFGLGLPATLVTALIGGTDSQGVIPGIPWNPDSEQAALDAVQKAGWTPITATQLGYDGKTDARGTFFGEKPGFTSAQVEILGKYDAQGQLTEIGIAFRGTSGPRESQISDSIGDVINDLLAALGPKDYAKNYADEAFGKLLADVAAFASAHGLSGEDVLVSGHSLGGLGVNSLADLSESKWGGFFKDANYIAYASPTQSETDKVLNIGYENDPVFRALDGSSFNLASVGVHDTQQDSATNNIVSFNDHYASTAWNLLPFSILNIPTWISHLPTGYGDGMTRVLDSKFYDLTHKDSTVIVANLSDPARANTWVQDLNRNAETHKGSTFIIGSDGNDLIQGGSGNDYLEGRDGNDTFRDAGGYNILLGGQGNNVLDLQQSVKNFDFANDGAGNLYIRDANGGISITRDIGSIVTKEPGFLWGLFKDDVTRSVTADGLKVGFQLTQYEASVKGGAGADTLKAHAGGDWLFGLDGNDHLLGGSGNDVFVGGAGNDLMESGGGYDTFLFSGAFGHDRVVGYQANDKLVFLGVEGVTANDNVRAHATVVGQDTVLTFGNDSVTLVGVGLDSLSSAGIVIA

>Pseudomonas sp. CR-611（AFP20145） I.3

MGVYDYKNFGTAESKALFSDAMAITLYSYHNLDNGFAVGYQHNGFGLGLPATLVTALIGGTDSQGVIPGIPWNPDSEKAALDAVQKAGWTPITASQLGYEGKTDARDTFFGEKAGYTSAQVEILGKHDAQGHLTEIGIAFRGTSGPREILIGDSIGDVINDLLAAFGPKDYAKNYVGEAFGNLMNDVVAFAKANGLTGKDVLVSGHSLGGLAVNSMADLSTSKWGGFFQYSNYIAYASPTQSSTDKVLNVGYENDPVFRALDGSTFTGASLGVHDAPKESATDNIVSFNDHYALAAWNVLPYSIVNIPTWISHLPTAYGDGMNRVIESKFYDLTSKNSTIIVANLSDPARANTWVQDLNRNAETHKGSTFIIGSDANDLIQGGSGNDYLEGRAGNDTFRDGGGYNILLGGSGNNTLELQKSVNTFDFANDGAGNLYIRDANGGISITRDIGSIVTKEPGFLWGLFKDDVTHSVTATGLKVGNNVTQYESSVKGTGGADTLKAKAGGDWLFGLDGNDHLIGGAGNDVFVGGAGNDLMESGGGADTFLFSGAFGQDRVVGYTANDKLVFLGVQGVLPNDDFRAHATAVGQDTVLKFGADSVTLVGVALNSLSADGIVIA

>Pseudomonas fluorescens（AAT48728） I.3

MGVYDYKNFGTAESKALFSDAMAITLYSYHNLDNGFAVGYQHNGFGLGLPATLVTALIGGTDSQGVIPGIPWNPDSEKAALDTVKKAGWTPITAAQLGYEGKTDARGTFFGEKAGYTSAQVEILGKYDAQGHLTEIGIAFRGTSGPREILIGDSIGDVINDLLAAFGPKDYAKNYVGEAFGNLMNDVVAFAKANGLSGKDVLVSGHSLGGLAVNSMADLSTSKWSGFFQDSNYIAYASPTQSSTDKVLNVGYENDPVFRALDGSTFTGASLGVHDAPKESATDNIVSFNDHYASAAWNVLPFSIVNIPTWISHLPTAYGDGMNRVIESKFYDLTSKDSTIIVANLSDPARANTWVQDLNRNAETHKGSTFIIGSDGNDLIQGGSGNDYLEGRAGNDTFRDGGGYNILLGGSGNNTLELQTSVNSFDFANDGAGNLYIRDANGGISITRDIGSIVTKEPGFLWGLFKDDVTHSVTAAGLKVGNNVTQYESSVKGTGGADTLKAKASGDWLFGLDGNDHLIGGAGNDVFVGGAGNDLLESGGGADTFLFSGAFGQDRVVGYTANDKLVFLGVQGVLPGDDLRAHATAVGQDTVLKFGADSVTLVGVALNSLSADGIVIA

>Bacillus subtilis(AAA22574) I.4

MKFVKRRIIALVTILMLSVTSLFALQPSAKAAEHNPVVMVHGIGGASFNFAGIKSYLVSQGWSRDKLYAVDFWDKTGTNYNNGPVLSRFVQKVLDETGAKKVDIVAHSMGGANTLYYIKNLDGGNKVANVVTVGGANRLTTGKALPGTDPNQKILYTSIYSSADMIVMNYLSRLDGARNVQIHGVGHIGLLYSSQVNSLIKEGLNGGGQNTN

>Bacillus pumilus(CAA02196) I.4

AEHNPVVMVHGIGGASYNFFSIKSYLATQGWDRNQLYAIDFIDKTGNNRNNGPRLSRFVKDVLDKTGAKKVDIVAHSMGGANTLYYIKNLDGGDKIENVVTIGGANGLVSSRALPGTDPNQKILYTSVYSSADLIVVNSLSRLIGARNILIHGVGHIGLLTSSQVKGYIKEGLNGGGQNTN

>Geobacillus thermocatenulatus(CAA64621) I.5

MMKGCRVMVVLLGLWFVFGLSVPGGRTEAASPRANDAPIVLLHGFTGWGREEMLGFKYWGGVRGDIEQWLNDNGYRTYTLAVGPLSSNWDRACEAYAQLVGGTVDYGAAHAAKHGHARFGRTYPGLLPELKRGGRVHIIAHSQGGQTARMLVSLLENGSQEEREYAKAHNVSLSPLFEGGHHFVLSVTTIATPHDGTTLVNMVDFTDRFFDLQKAVLKAAAVASNVPYTSQVYDFKLDQWGLRRQPGESFDHYFERLKRSPVWTSTDTARYDLSIPGAEKLNQWVQASPNTYYLSFSTERTHRGALTGNYYPELGMNAFSAVVCAPFLGSYRNEALGIDDRWLENDGIVNTVSMNGPKRGSSDRIVPYDGTLKKGVWNDMGTCNVDHLEVIGVDPNPSFDIRAFYLRLAEQLASLRP

>Staphylococcus hyicus(CAA26602) I.5

MKETKHQHTFSIRKSAYGAASVMVASCIFVIGGGVAEANDSTTQTTTPLEVAQTSQQETHTHQTPVTSLHTATPEHVDDSKEATPLPEKAESPKTEVTVQPSSHTQEVPALHKKTQQQPAYKDKTVPESTIASKSVESNKATENEMSPVEHHASNVEKREDRLETNETTPPSVDREFSHKIINNTHVNPKTDGQTNVNVDTKTIDTVSPKDDRIDTAQPKQVDVPKENTTAQNKFTSQASDKKPTVKAAPEAVQNPENPKNKDPFVFVHGFTGFVGEVAAKGENHWGGTKANLRNHLRKAGYETYEASVSALASNHERAVELYYYLKGGRVDYGAAHSEKYGHERYGKTYEGVLKDWKPGHPVHFIGHSMGGQTIRLLEHYLRFGDKAEIAYQQQHGGIISELFKGGQDNMVTSITTIATPHNGTHASDDIGNTPTIRNILYSFAQMSSHLGTIDFGMDHWGFKRKDGESLTDYNKRIAESKIWDSEDTGLYDLTREGAEKINQKTELNPNIYYKTYTGVATHETQLGKHIADLGMEFTKILTGNYIGSVDDILWRPNDGLVSEISSQHPSDEKNISVDENSELHKGTWQVMPTMKGWDHSDFIGNDALDTKHSAIELTNFYHSISDYLMRIEKAESTKNA

>Staphylococcus aureus(AAA26633) I.5

MLRGQEERKYSIRKYSIGVVSVLAATMFVVSSHEAQASEKTSTNAAAQKETLNQPGEQGNAITSHQMQSGKQLDDMHKENGKSGTVTEGKDTLQSSKHQSTQNSKTIRTQNDNQVKQDSERQGSKQSHQNNATNNTERQNDQVQNTHHAERNGSQSTTSQSNDVDKSQPSIPAQKVIPNHDKAAPTSTTPPSNDKTAPKSTKAQDATTDKHPNQQDTHQPAHQIIDAKQDDTVRQSEQKPQVGDLSKHIDGQNSPEKPTDKNTDNKQLIKDALQAPKTRSTTNAAADAKKVRPLKANQVQPLNKYPVVFVHGFLGLVGDNAPALYPNYWGGNKFKVIEELRKQGYNVHQASVSAFGSNYDRAVELYYYIKGGRVDYGAAHAAKYGHERYGKTYKGIMPNWEPGKKVHLVGHSMGGQTIRLMEEFLRNGNKEEIAYHKAHGGEISPLFTGGHNNMVASITTLATPHNGSQAADKFGNTEAVRKIMFALNRFMGNKYSNIDLGLTQWGFKQLPNESYIDYIKRVSKSKIWTSDDNAAYDLTLDGSAKLNNMTSMNPNITYTTYTGVSSHTGPLGYENPDLGTFFLMATTSRIIGHDAREEWRKNDGVVPVISSLHPSNQPFVNVTNDEPATRRGIWQVKPIIQGWDHVDFIGVDFLDFKRKGAELANFYTGIINDLLRVEATESKGTQLKAS

>Propionibacterium acnes(CAA67627) I.6

MKINARFAVMAASVAVLMAAAPIAQAATSPGDIHPLVQAAHSPDGIPGNGVGPEFHTSSMARSYSEKHLGVAPRGVNDFSCKVKPGDRPVILIPGTGGNAFATWSFYGPHLAHEGYCVYTFTTNVPVGILDEGWGFTGDVRASAQALGAFVDRVRKATGSEKVDFVGHSQGGGILPNAYIKMYGGASKVDKLIGLVAANHGTTAVGLDKLVDGLPEAVKDFLSTWSYDHNMEAYGQQLKGSALMQQVYRDGDTVPGIAYTVISTRLDMTVTPYTQAFLKGAKNMTVQDACPLDAYGHGRLPYDPVAYQMVLNALDPNHPREISCTWRPRVLPVSTTDAA

>Streptomyces cinnamoneus(AAB71210) I.6

MRLRRTVRFLIAAAATAALGLTGLSAPAAASVLDVPPGGANDWSCKPDSAHPQPVVLVNGTFKLMAENWSKLSPKLKEAGYCVFAFNYGHFETDPIPESAAELRDFVEAVRGATGAAKVDIVGHSQGGMLPRYYVKFLGGADKVDDLVGIVPSNHGTKNPLAIPAGWTFCPSCVDQQAGSELLQKLNAGDETPAGPDYTVITTRYDEVVIPYASALLTGDKEHLTNVVLQDKCPLDLYMHDQATKDPVVAQWVLDALARKGPADPGFQPRCLGGA

**FIG 4 Multiple sequence alignment between Pflip1 and other closely related lipolytic enzymes**

>ABA72315

MSQDSATRYPLVLVPGMLGFIRLVLYPYWYGIIKALRRGGATVIAVQVSPLNSTEVRGEQLLTRIDEILRETGAAKVNLFGHSQGSLTARYAAAKRPDLVASVTSVAGPNHGSELADYLAKYYPADSAKGRILEALLRSVGWLMALLETGYHGPKLPVDIHASHHSLTTEGVALFNQLYPQGLPQTWGGHGPEEVNGVRYYSWSGTLQPGKTDRGGNLFDGTNRSCRLFAKTFVREPGQCDGMVGRYSSHLGTVIGDDYPMDHFDIVNQSLGLVGKGADPVRLFVEHAARLKAAGV

>1EX9

STYTQTKYPIVLAHGMLGFDNILGVDYWFGIPSALRRDGAQVYVTEVSQLDTSEVRGEQLLQQVEEIVALSGQPKVNLIGHSHGGPTIRYVAAVRPDLIASATSVGAPHKGSDTADFLRQIPPGSAGEAVLSGLVNSLGALISFLSSGSTGTQNSLGSLESLNSEGAARFNAKYPQGIPTSACGEGAYKVNGVSYYSWSGSSPLTNFLDPSDAFLGASSLTFKNGTANDGLVGTCSSHLGMVIRDNYRMNHLDEVNQVFGLTSLFETSPVSVYRQHANRLKNASL

>AAC15585

MSQSTATRYPLVLVPGMLGFIRLLLYPYWYGIIKALRRGGATVIAVQVSPLNSTEVRGEQLLARIDEILRETGAARVNLFGHSQGSLTARYAAAKRPDLVASVTSVAGPNHGSELADYLQQHYPANTAKGRLLEALLRLIGWLMARLETGYHGPKLPVDIHASHQSLTREGVALFNQRYPQGLPETWGGQGPEVVNGVRYYSWSGTLQPGKTDRGGNLFDGTNRSCRLFAKTFVREPGQCDGMVGRYSSHLGTVIGDDYPLDHFDIVNQSLGLVGKGADPVRLFVEHAARLKAAGL

>AEV60646

MLTPLDHKGVAMSQRCATRYPLVLVPGMLGFIRLVLYPYWYGIVSALRRGGAVVVAVKVSPLHSSEVRGEQLLARIEEILRQTGAQKVNLIGHSQGSLTARYAAAKRPDLVASVTSVAGTNHGSELADYLQLHYPADSAKGRVLSAVLRLINALMSLLDTGYRGPKLPVDVHASHASLTTAGVGVFNQRYPQGLPVTWGGNGPEEVNGVRYYSWSGTLQPGKTDKGRNLFDGTNRSCRLFARTFVREAGQCDGMVGRYSSHLGTVIGDDYPLDHFDIVNQSLGLVGKGAEPIRLFVEHAERLKAAGV

>WP_015093259

MSQGSAPRYPLVLVPGMLGFIRLVLYPYWYGIVEVLRHGGAVVFAVQVSPLNSNEVRGEQLLARIDEILRETGADKVNLIGHSQGSLTARYAAAKRPDLVASVTSVAGPNHGSELADYLHQHYPHDSLRGRLLCFLLRMIGTLMGWLDTGYHGPKLPVDIHASHHALTTAGVALFNQRYPQGLPETWGGHGPEEVNGVRYYSWSGTLQPGITDRGRNLLDGTNRSCRLFAKTFVREAGHCDGMVGRYSSHLGTVIGDEYPLDHFDIVNQSLGLVGKGAEPIRLFVEHAARLSATGL

>CAA32193

MDDSVNTRYPILLVHGLFGFDRIGSHHYFHGIKQALNECGASVFVPIISAANDNEARGDQLLKQIHNLRRQVGAQRVNLIGHSQGALTARYVAAIAPELIASVTSVSGPNHGSELADRLRLAFVPGRLGETVAAALTTSFSAFLSALSGHPRLPQNALNALNALTTDGVAAFNRQYPQGLPDRWGGMGPAQVNAVHYYSWSGIIKGSRLAESLNLLDPLHNALRVFDSFFTRETRENDGMVGRFSSHLGQVIRSDYPLDHLDTINHMARGSAGASTR

>CAC07191

MDDSVNTRYPILLVHGLFGFDRIGSHHYFHGIKQALNECGASVFVPIISAANDNEARGDQLLKQIHNLRRQVGAQRVNLIGHSQGALTARYVAAIAPELIASVTSVSGPNHGSELADRLRLAFVPGRLGETVAAALTTSFSAFLSALSGHPRLPQNALNALNALTTDGVAAFNRQYPQGLPDRWGGMGPAQVNAVHYYSWSGIIKGSRLAESLNLLDPLHNALRVFDSFFTRETRENDGMVGRFSSHLGQVIRSDYPLDHLDTINHMARGSRRRINPVELYIEHAKRLKEAGL
